# Supply and demand of creatine and glycogen in broiler chicken embryos

**DOI:** 10.3389/fphys.2023.1079638

**Published:** 2023-01-24

**Authors:** Jonathan Dayan, Tal Melkman-Zehavi, Naama Reicher, Ulrike Braun, Vivienne Inhuber, Sameer J. Mabjeesh, Orna Halevy, Zehava Uni

**Affiliations:** ^1^ Department of Animal Science, Robert H. Smith Faculty of Agriculture, Food and Environment, The Hebrew University of Jerusalem, Rehovot, Israel; ^2^ AlzChem Trostberg GmbH, Trostberg, Germany

**Keywords:** incubation, chicken, embryo, creatine, glycogen

## Abstract

Optimal embryonic development and growth of meat-type chickens (broilers) rely on incubation conditions (oxygen, heat, and humidity), on nutrients and on energy resources within the egg. Throughout incubation and according to the embryo’s energy balance, the main energy storage molecules (creatine and glycogen) are continuously utilized and synthesized, mainly in the embryonic liver, breast muscle, and the extraembryonic yolk sac (YS) tissue. During the last phase of incubation, as the embryo nears hatching, dynamic changes in energy metabolism occur. These changes may affect embryonic survival, hatchlings’ uniformity, quality and post hatch performance of broilers, hence, being of great importance to poultry production. Here, we followed the dynamics of creatine and glycogen from embryonic day (E) 11 until hatch and up to chick placement at the farm. We showed that creatine is stored mainly in the breast muscle while glycogen is stored mainly in the YS tissue. Analysis of creatine synthesis genes revealed their expression in the liver, kidney, YS tissue and in the breast muscle, suggesting a full synthesis capacity in these tissues. Expression analysis of genes involved in gluconeogenesis, glycogenesis, and glycogenolysis, revealed that glycogen metabolism is most active in the liver. Nevertheless, due to the relatively large size of the breast muscle and YS tissue, their contribution to glycogen metabolism in embryos is valuable. Towards hatch, post E19, creatine levels in all tissues increased while glycogen levels dramatically decreased and reached low levels at hatch and at chick placement. This proves the utmost importance of creatine in energy supply to late-term embryos and hatchlings.

## Introduction

The phase of embryonic development has great importance to the production chain of meat-type chickens (broilers) as the 21-day of incubation constitutes more than a third of the broiler lifespan, starting with incubation and ending with marketing at 35–42 days (Havenstein et al., 2003a; Havenstein et al., 2003b; Zuidhof et al., 2014; Givisiez et al., 2020). During the period of incubation, in order to ensure optimal development and growth, avian embryos depend on levels of oxygen, heat and humidity together with the supply of nutrients and energy (Decuypere et al., 2001; Moran, 2007; De Oliveira et al., 2008). Embryos utilize the nutrients within the egg to form high energy-value compounds such as glycogen and creatine with the final aim of ATP production (Christensen et al., 1999; 2000; 2001; Moran, 2007).

Glycogen is the main storage form of glucose in tissues, its high-energetic value is derived from the potential to release glucose and the production of ATP by aerobic respiration or by anaerobic glycogen breakdown (glycolysis). Two key enzymes are involved in glycogen metabolism; glycogen synthase (GYS), a rate limiting enzyme in glycogen synthesis, and glycogen phosphorylase L (PYGL), which catalyzes the release of glucose-1-phosphate from glycogen (Blaxter, 1989; Scanes, 2022). During most of incubation period, up to embryonic day (E) 19, gas exchange is achieved through chorioallantoic respiration and oxygen is sufficient to support aerobic processes of energy metabolism and lipid oxidation (Tazawa et al., 1983; Decuypere et al., 2001; Moran, 2007). Along this period, sufficient glucose is produced, and the unutilized glucose is funneled to storage, i.e., glycogen synthesis in the embryonic liver, muscles, and the extraembryonic yolk sac (YS) tissue (Romanoff, 1960; Romanoff, 1967; Noble and Cocchi, 1990; Speake et al., 1998; Yadgary and Uni, 2012; Wong and Uni, 2021). Post E19, prior to the initiation of the hatching process, oxygen levels become limiting. At this phase, energy production in the embryo switches from lipid oxidation to anaerobic catabolism, by the breakdown of glycogen to glucose and by gluconeogenesis with the breakdown of amino acids for glucose and ATP production. The latter is apparent with the activation of two key gluconeogenic enzymes; fructose-1,6-bisphosphatase 1 (FBP1), which catalyzes the hydrolysis of fructose 1,6-bisphosphate to fructose 6-phosphate, and glucose-6-phosphatase 2 (G6PC2), which catalyzes the hydrolysis of glucose-6-phosphate to glucose and inorganic phosphate, leading to the release of glucose into the bloodstream. By the time the embryo hatches, almost all glycogen is exploited, and its stores are depleted (Uni and Ferket, 2004; De Oliveira et al., 2008; Yadgary and Uni, 2012).

Creatine, another high-energy value molecule, is deposited by the hen in the egg compartments (yolk and albumen) where it is available to the embryo (Reicher et al., 2020). However, as only small amounts are deposited, the embryo relies also on *de novo* synthesis of creatine during incubation (Walker, 1979). Creatine synthesis involves two main steps driven by two enzymes. The first enzyme, L-arginine: glycine amidinotransferase (AGAT), catalyzes the reaction between glycine and arginine to create ornithine and guanidinoacetic acid (GAA). The second enzyme is guanidinoacetate N-methyltransferase (GAMT), which is responsible for GAA methylation *via* S-adenosylmethionine (SAM) to form creatine. Once inside the cells, as shown in rats and mice, creatine is phosphorylated into phosphocreatine, initiating the conversion of ADP into ATP during its dephosphorylation. Thus, creatine functions as an energy storage molecule, which can generate ATP on demand (Bessman and Carpenter, 1985; Wyss, 2000; Da Silva et al., 2009). Additionally, unlike glycogen or fat, no metabolic breakdown is needed for ATP production from the creatine/phosphocreatine system, making it the fastest way of ATP provision. In chicken embryos and hatchlings, creatine was shown to play a major role in energy metabolism, with the potential to promote their energetic status and the post-hatch performance (Murakami et al., 2014; Zhang et al., 2016). However, little is known about the dynamics of creatine during chicken embryogenesis and its relation to glycogen stores, as well as to the energetic status of late-term embryos and hatchlings. Moreover, previous studies have not shown an inclusive comparison of glycogen and creatine dynamics simultaneously in the liver, YS tissue, and breast muscle.

This study examined creatine and glycogen dynamics from E11 until hatch and up to chick placement at the farm. Embryos and hatchlings were sampled and examined for; (1) creatine and glycogen levels in the liver, YS tissue, and breast muscle, (2) levels of blood glucose and ketones, and (3) the expression levels of genes encoding for enzymes involved in creatine synthesis in the liver, kidney, YS tissue and breast muscle (AGAT and GAMT), along with genes that are encoding for gluconeogenic enzymes (FBP1 and G6PC2), and enzymes responsible for glycogen synthase (GYS), and glycogenolysis (PYGL).

## Materials and methods

### Eggs, incubation and sampling procedure

Fertile eggs (*n* = 100; mean weight = 70.04 g, SD = 2.19 g) from 40-week-old broiler hens (Cobb 500) were purchased from a commercial breeder farm (Y. Brown and Sons Ltd., Hod Hasharon, Israel). Eggs were incubated in a Petersime hatchery at the Faculty of Agriculture of the Hebrew University under standard conditions (37.8°C and 56% relative humidity). On embryonic day (E) 10, eggs were candled, unfertilized eggs and dead embryo eggs were removed. Tissue sampling was performed at E11, E13, E15, E17, E19, at hatch (actual time of hatch at the hatchery, 480–483 h in incubation) and at chick placement (31–34 h post hatch, prior to the exposure to feed at the farm). At each sampling day, six embryos/hatchlings were randomly selected, euthanized by cervical dislocation and the following parameters were recorded: embryo yolk free body mass (YFBM), yolk content weight, YS tissue weight (according to Yadgary et al., 2010), liver weight and breast muscle weight. The relative weights of liver, breast muscle and YS tissue were calculated as percent of YFBM. Blood glucose and ketone levels were also recorded. In addition, liver, YS tissue and breast muscle samples were collected for creatine and glycogen content determination and for the evaluation of expression levels of genes involved in creatine synthesis, gluconeogenesis, glycogenesis, and glycogenolysis. Kidney samples were collected for the evaluation of expression of creatine synthesis. Immediately after collection, tissue samples were placed in liquid nitrogen and kept under −80^o^c until further processing.

### Creatine and glycogen content determination

Liver, YS tissue and breast muscle samples were lyophilized and later examined for their creatine and glycogen content by Swiss-BioQuant-AG (Reinach, Switzerland). The concentrations of creatine and glycogen (mg/g dry weight tissue) were determined, and the total amounts (mg) in each tissue were calculated. Due to insufficient material load, breast muscle samples were analyzed only at E15, E17, E19, hatch and chick placement.

### Glucose and ketones measurements

Blood glucose and ketones levels were obtained directly from the jugular vein of embryos and determined using FreeStyle Optimum Gluco/Keto meter and test strips (Abbott Diabetes Care Ltd., Witney Oxon, United Kingdom) according to the manufacturer’s instructions.

### RNA isolation, cDNA synthesis and determination of mRNA abundance by real-time PCR

Total RNA was isolated from 100 mg of tissue (from YS tissue, liver, kidney and breast muscle) using TRI-Reagent (Sigma-Aldrich, St. Louis, MO) according to the manufacturer’s protocol. RNA concentration was determined using a Nano Drop ND-1000 instrument (Thermo Fisher Scientific, Wilmington, DE). Total RNA was treated with DNase using a Turbo DNA-free Kit according to the manufacturer’s protocol (Ambion; Thermo Fisher Scientific, Wilmington, DE). cDNA was created from 1 µg of DNA-free RNA using the qPCRBIO cDNA synthesis kit according to the manufacturer’s protocol (PCRBIOSYSTEMS, London, United Kingdom). Relative mRNA expression was evaluated using gene-specific primers (Table 1) of genes involved in creatine synthesis [arginine-glycine amidinotransferase (AGAT), guanidinoacetate N-methyltransferase (GAMT)], gluconeogenesis [fructose-1,6-bisphosphatase 1 (FBP1), glucose-6-phosphatase 2 (G6PC2)], glycogenesis [glycogen synthase (GYS)], and glycogenolysis [glycogen phosphorylase L (PYGL)] and the housekeeping genes; cytoskeletal protein (β-actin) and hypoxanthine phosphoribosyl transferase 1 (HPRT). Primer sequences were designed using Primer-BLAST software (Ye et al., 2012) based on published cDNA sequences where available and purchased from Sigma-Aldrich (Rehovot, Israel). PCR products were validated by gel electrophoresis in 1.5% agarose gel. Real-time qPCR reactions were conducted in triplicate in a Roche Light cycler 96 (Roche Molecular Systems, Inc., Pleasanton, CA). Each reaction (20 µL) included 3 µL of cDNA sample diluted 1:20 in ultrapure water (UPW, Biological Industries, Beit HaEmek, Israel), 4 µM of each primer, and Platinum SYBR Green qPCR super mix-UDG (Thermo Fisher Scientific, Wilmington, DE). Reaction conditions: preincubation at 95°C for 60°s, followed by 40 cycles of a 2-step amplification cycle of 95°C for 10°s and 60°C for 30°s, ended with a melting curve generated by the following conditions: 95°C for 60°s, 65°C for 60°s, and 97°C for 1 s. Relative mRNA expression was calculated by subtracting the geometric mean of cycle threshold (Ct) values of β-Actin and HPRT reference genes from sample Ct (Pfaffl, 2007).

**TABLE 1:** Primers used for real-time PCR gene expression analysis.

Target^a^	Accession number	Primer F (5′-3′)	Primer R (5′-3′)	Amplicon	References
AGAT	NM204745.1	ACA​TCT​TGC​ACC​TGA​CTA​CCG	ACA​GTG​GGT​GAT​CAT​CAG​GAA	206	Reicher et al. (2020)
GAMT	XM015299974.2	ACA​CAA​GGT​GGT​GCC​ACT​GA	CGA​GGT​GAG​GTT​GCA​GTA​GG	199	Reicher et al. (2020)
GYS1	AB090806	GAT​GAA​ACG​CGC​CAT​CTT​CG	CGC​ACG​AAC​TCC​TCG​TAG​TC	213	Primer Blast
GYS2	XM_015291547.2	CAT​CTG​TAC​ACT​GTG​CCC​ATG​TG	TTT​GGA​GTG​ACA​ACA​TCA​GGA​TTT	92	Yadgary and Uni. (2012)
PYGL	NM_204392.2	CCG​TCC​TCC​ATG​TTT​GAT​GTG	TCT​TGA​TGC​GGT​TGT​ACA​TGG​T	100	Yadgary and Uni. (2012)
FBP1	NM_001278048.1	TTC​CAT​TGG​GAC​CAT​ATT​TGG	ACC​CGC​TGC​CAC​AAG​ATT​AC	100	Yadgary and Uni. (2012)
G6PC2	NC_052538.1	CCT​TCA​CAG​ACT​GAC​ATG​GTC​ATT​A	ATG​AGG​GAA​ATG​TGT​TGC​TAT​GAA​T	100	Yadgary and Uni. (2012)
HPRT	NM_204848	AAG​TGG​CCA​GTT​TGT​TGG​TC	GTA​GTC​GAG​GGC​GTA​TCC​AA	110	Boo et al. (2020)
β-actin	NM_205518.1	AAT​GGC​TCC​GGT​ATG​TGC​AA	GGC​CCA​TAC​CAA​CCA​TCA​CA	112	Primer Blast

^a^ Arginine-glycine amidinotransferase (**AGAT**), catalyzes the reaction between glycine and arginine to create GAA; guanidinoacetate N-methyltransferase (**GAMT**), catalyzes the methylation of GAA, to create creatine; glycogen synthase (**GYS**), catalyzes the rate-limiting step in the synthesis of glycogen (GYS1 is the muscle variant and GYS2 is expressed in other tissues); glycogen phosphorylase L (**PYGL**), catalyzes the release of glucose-1-phosphate from glycogen; fructose-1,6-bisphosphatase 1 (**FBP1**), catalyzes the hydrolysis of fructose 1,6-bisphosphate to fructose 6-phosphate; glucose-6-phosphatase 2 (**G6PC2**), catalyzes the hydrolysis of glucose-6-phosphate, allowing the release of glucose into the bloodstream; β-actin is a housekeeping cytoskeletal protein (**β-actin**), and hypoxanthine phosphoribosyl transferase 1 (**HPRT**) is an enzyme in the purine synthesis in salvage pathway, also a housekeeping gene.

### Statistical analysis

Data of creatine and glycogen concentrations in tissues and data of blood glucose and ketone levels were subjected to one-way ANOVA analysis with the embryonic day (age) as main effect. Data of creatine and glycogen amount and of gene expression in tissues were subjected to two-way ANOVA analysis with the embryonic day (age), tissue and their interaction as the main effects. Differences between means were tested by using Tukey’s HSD test and considered significantly different with *p*-value lower than or equal to 0.05 (*p* ≤ 0.05). Values are presented as mean ± standard error mean (SEM). All statistical analyses were carried out using JMP-pro 16 software (SAS Institute Inc., Cary, NC).

## Results

### Creatine and glycogen levels in the breast muscle, liver and YS tissue of chicken embryos

**FIGURE 1:**
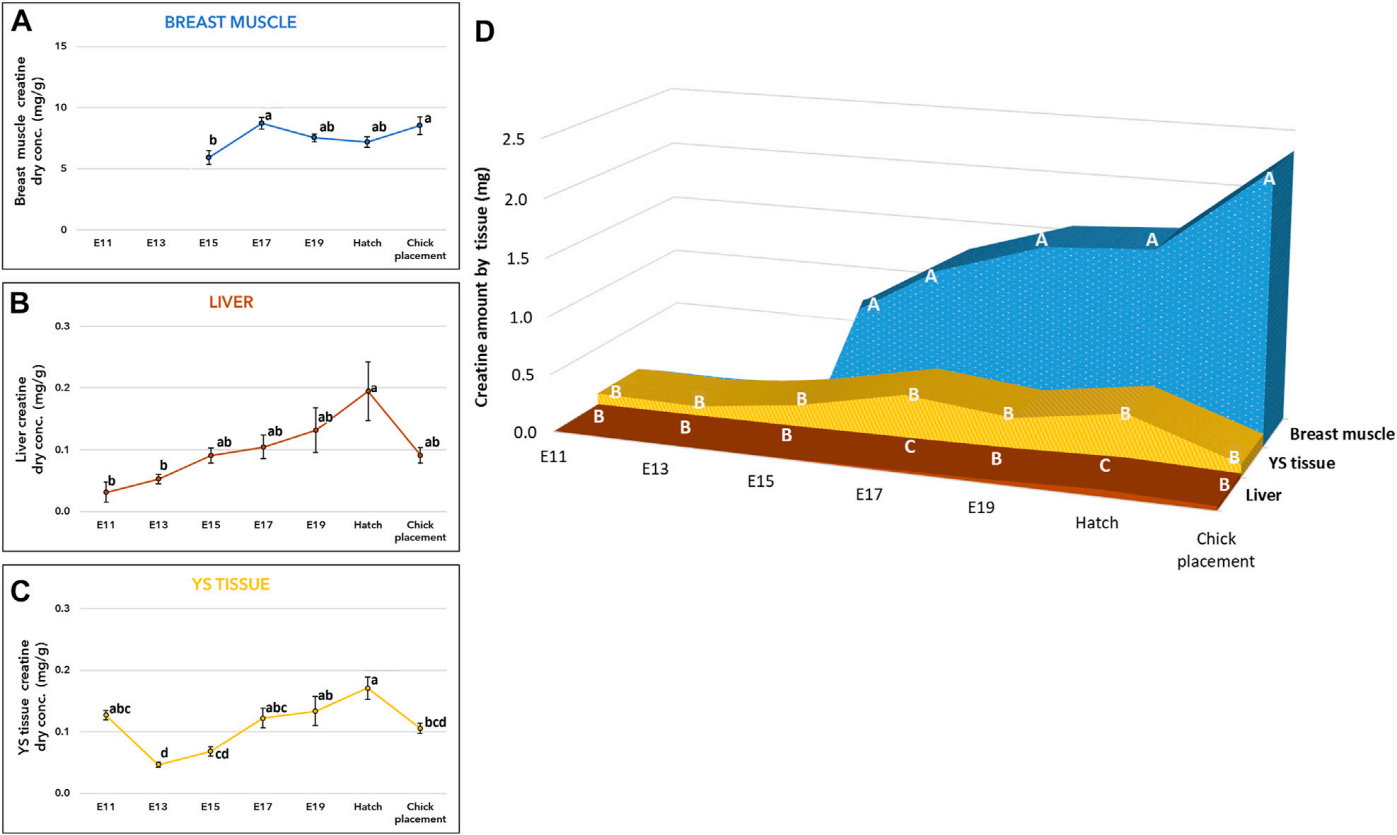
Creatine levels from E11 until hatch and at chick placement **(A–C)** Dry weight concentration (mg/g) and **(D)** total tissue amount (mg) of Yolk sac (YS) tissue, liver, and breast muscle **(A–C)** The lower-case letters denote for means significantly different between days within each organ, as derived from a one-way ANOVA Tukey’s HSD test **(D)** The capital letters denote for means significantly different between organs within each day as derived from a two-way ANOVA followed by Tukey’s HSD test. *p* ≤ 0.05 *n* = 6 per day.

Measurements of creatine concentration (mg/g of dry tissue) in the breast muscle showed a significant increase by 47% (*p*-value, *p* = 0.0009) from embryonic day (E) 15 to E17 (Figure 1A, and Supplementary Table S1). Then, throughout E19, hatch and chick placement, creatine levels did not differ significantly between consecutive days. The liver and yolk sac (YS) tissue exhibited similar patterns (Figures 1B, C, and Supplementary Table S1), as creatine concentration increased by 3.8-fold and 3.4-fold respectively from E13 up to the highest value at hatch (*p* < 0.0001). Different from the breast muscle, creatine concentration decreased in the liver and YS tissue at chick placement by 2-fold and 1.5-fold, respectively. The total amount of creatine in each organ (Figure 1D and Supplementary Table S2) was calculated by multiplying the total dry tissue weight (Suppl. Table 6) by the concentration of creatine (Figures 1A–C and Supplementary Table S1). A two-way ANOVA test revealed a significant interaction between the effects of age and tissue (*p* < 0.0001). This was mainly due to the breast muscle tissue, as its creatine amount showed a constant elevation pattern unlike the liver and YS tissue. Results also show that creatine is stored mainly in the breast muscle, as its amount was 3-fold higher at hatch and 15-fold higher at chick placement compared with combined amounts of the liver and YS tissue. It should be noted that compared to the liver, the YS tissue had higher amounts of creatine throughout incubation, significantly higher on E17 and at hatch (Figure 1D and Supplementary Table S2).

**FIGURE 2:**
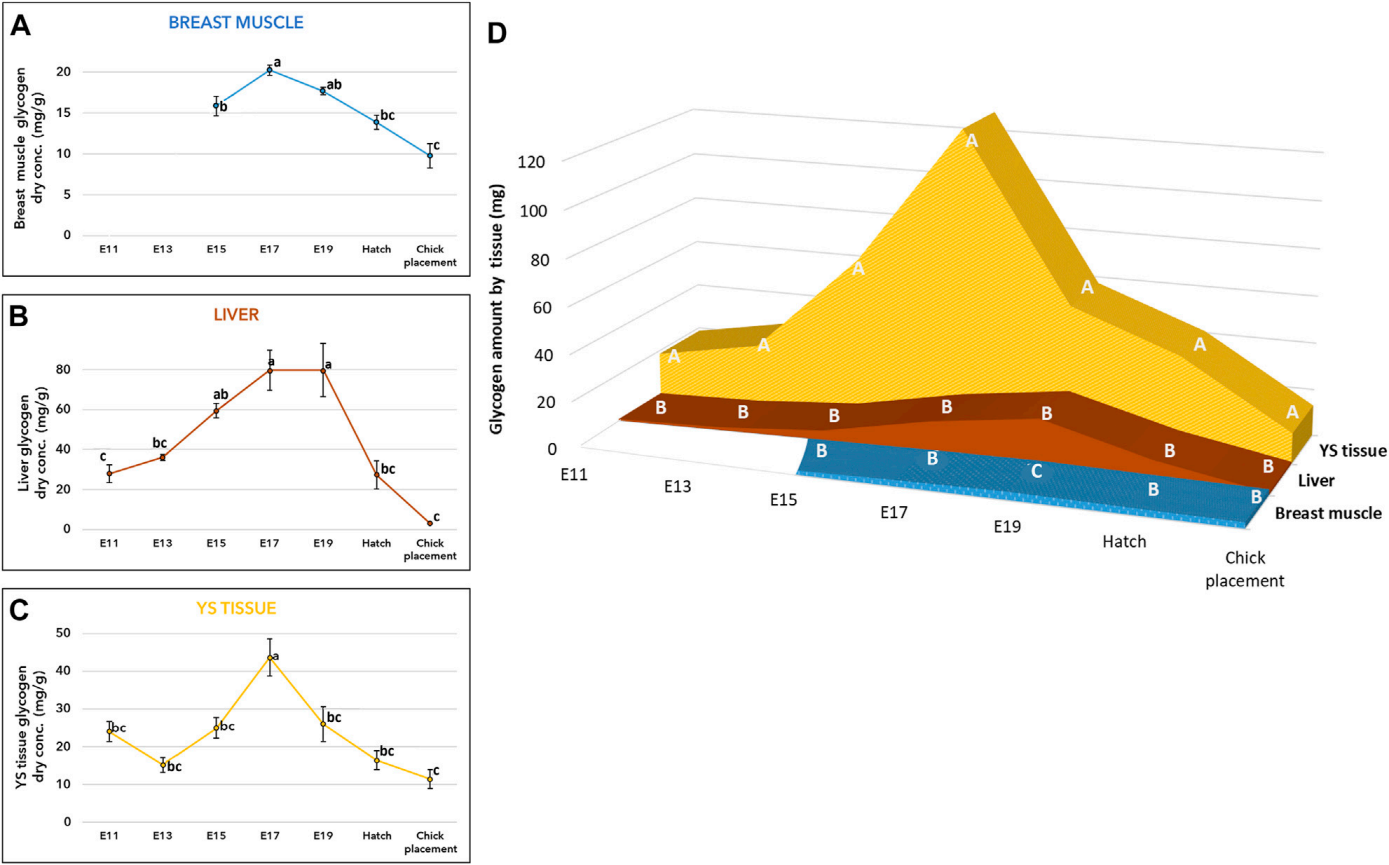
Glycogen levels from E11 until hatch and at chick placement **(A–C)** Dry weight concentration (mg/g) and **(D)** total tissue amount (mg) of YS tissue, liver, and breast muscle **(A–C)** The lower-case letters denote for means significantly different between days within each organ, as derived from a one-way ANOVA Tukey’s HSD test **(D)** The capital letters denote for means significantly different between organs within each day as derived from a two-way ANOVA followed by Tukey’s HSD test. (*p* ≤ 0.05), *n* = 6 per day.

As for glycogen, its concentration (mg/g of dry tissue) exhibited similar patterns in the breast muscle, liver, and YS tissue (Figures 2A–C, and Supplementary Table S1). Towards E17 an increase in glycogen levels was observed in all tissues, then towards hatch, glycogen levels were significantly decreased reaching the lowest levels at chick placement (*p* < 0.0001). The total amount of glycogen in each organ (Figure 2D and Supplementary Table S1) was also calculated by multiplying the total dry tissue weight (Supplementary Table S6) by the concentration in tissues (Figures 2A–C and Supplementary Table S1). A two-way ANOVA test revealed a significant interaction between the effects of age and tissue (*p* < 0.0001). The interaction was mainly due to the YS tissue, as its glycogen amount showed the most dynamic pattern, with a rapid increase up to E17 followed by a sharp decrease. In addition, throughout incubation the YS tissue had the highest amount of glycogen, as it was 27-fold higher on E11 compared to the liver and 3.3-fold higher at hatch and at chick placement, compared to the combined amount in the liver and breast muscle (Figure 2D and Supplementary Table S2).

### Blood glucose and ketones levels of chicken embryos

**FIGURE 3:**
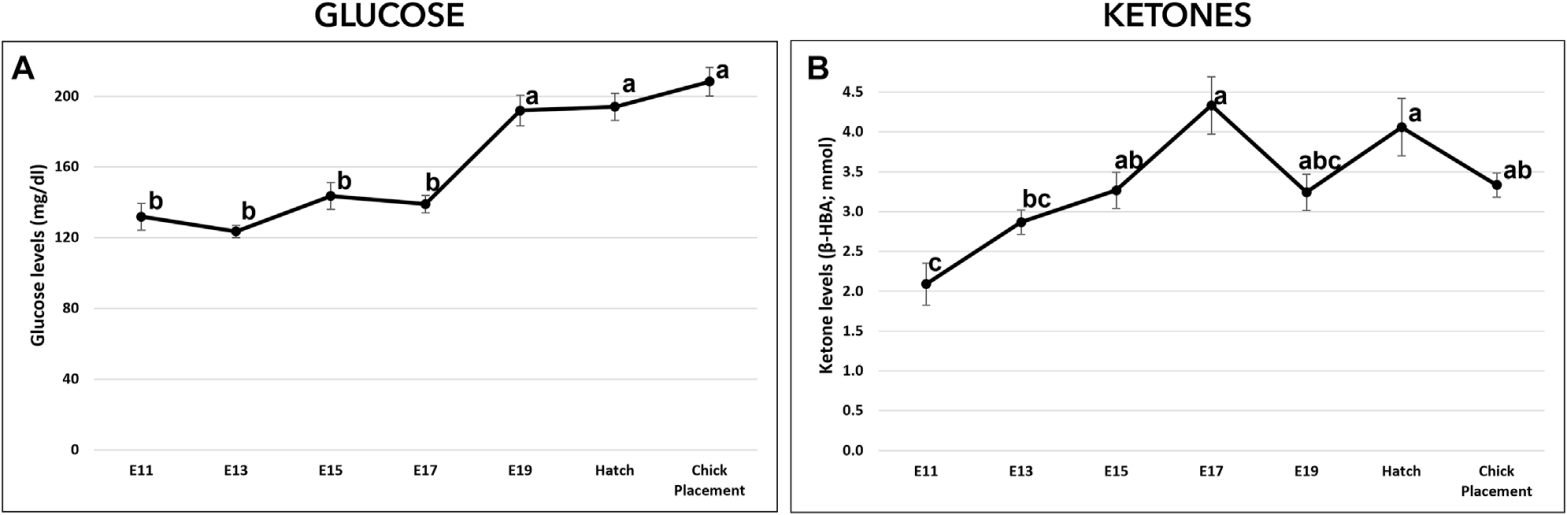
Glucose and ketones levels from E11 until hatch and at chick placement. **(A)**-Glucose (mg/di) and **(B)**-ketones ((3-HBA; mmol) were measured by FreeStyle Optimum Gluco/Keto meter. The lower-case letters denote for means significantly different between days, as derived from a one-way ANOVA Tukey’s HSD test (*p* < 0.05), *n* = 6 per day.

Glucose (mg/dL) and ketones (β-HBA; mmol/L) levels were measured from E11 to chick placement and exhibited altered patterns. From E11 to E17, glucose levels were relatively constant ranging between values of 123.5–143.6 mg/dL (Figure 3A). From E19 and until hatch and chick placement, blood glucose levels increased significantly by 38% (*p* < 0.0001), reaching values of 191.9–208.3 mg/dL. Ketone levels (Figure 3B) showed a dynamic pattern, starting on E11 with the lowest level (2.09 mmol), increasing gradually up to the highest value on E17 (4.33 mmol/L; *p* < 0.0001). On E19, ketones level decreased to 3.24 mmol/L while at hatch levels increased again to 4.06 mmol/L.

### Creatine synthesis genes in the breast muscle, kidney, liver, and YS tissue of chicken embryos

**FIGURE 4:**
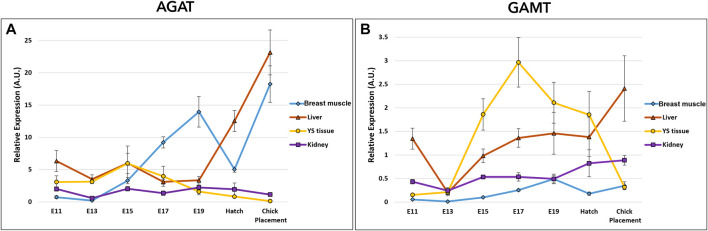
Dynamic expression of genes involved in creatine synthesis **(A)** Arginine-glycine amidinotransferase (AGAT), catalyzes the reaction between glycine and arginine to create GAA **(B)** Guanidinoacetate N-methyltransferase (GAMT), responsible for the methylation of GAA to create creatine. Expression analysis of AGAT and GAMT by a two-way ANOVA followed by Tukey’s HSD test revealed significant interactions between the effects of age and tissue. (*p* < 0.05), *n* = 6 per day.

The potential of creatine *de novo* synthesis in the tissues was evaluated by the examination of arginine-glycine amidinotransferase (AGAT), and guanidinoacetate N-methyltransferase (GAMT) (Figure 4 and Supplementary Table S3). Expression analysis of AGAT and GAMT by a two-way ANOVA test revealed significant interactions between the effects of age and tissue (*p* < 0.0001). The interaction was mainly associated with the varying expression patterns in tissues. In the breast muscle, AGAT expression showed a dynamic pattern, as it was increased from E11 to E19, followed by a decrease at hatch, then, sharply increased again reaching the highest level at chick placement. GAMT expression in the breast muscle also showed a dynamic pattern, increasing from E11 to E19 followed by a decrease at hatch. In the kidney, expression of AGAT remained stable and was not significantly different between consecutive embryonic days, however, GAMT expression increased gradually from E13 along the second half of incubation, reaching highest values at hatch and at placement. The liver and YS tissue showed alternating expression patterns as liver AGAT expression increased towards hatch and chick placement, and YS tissue AGAT expression decreased. As for GAMT expression, in the liver significantly different values were found on E13 as levels were decreased, while at chick placement levels were significantly increased. In the YS tissue GAMT expression increased up to E17, followed by a decrease up to chick placement. Altogether, results demonstrate a shift in expression levels of AGAT and GAMT, decreasing in the extraembryonic YS tissue towards hatch and placement, and increasing in the embryonic liver, breast muscle and kidney.

### Gluconeogenic genes in the liver, YS tissue, and breast muscle of chicken embryos

**FIGURE 5:**
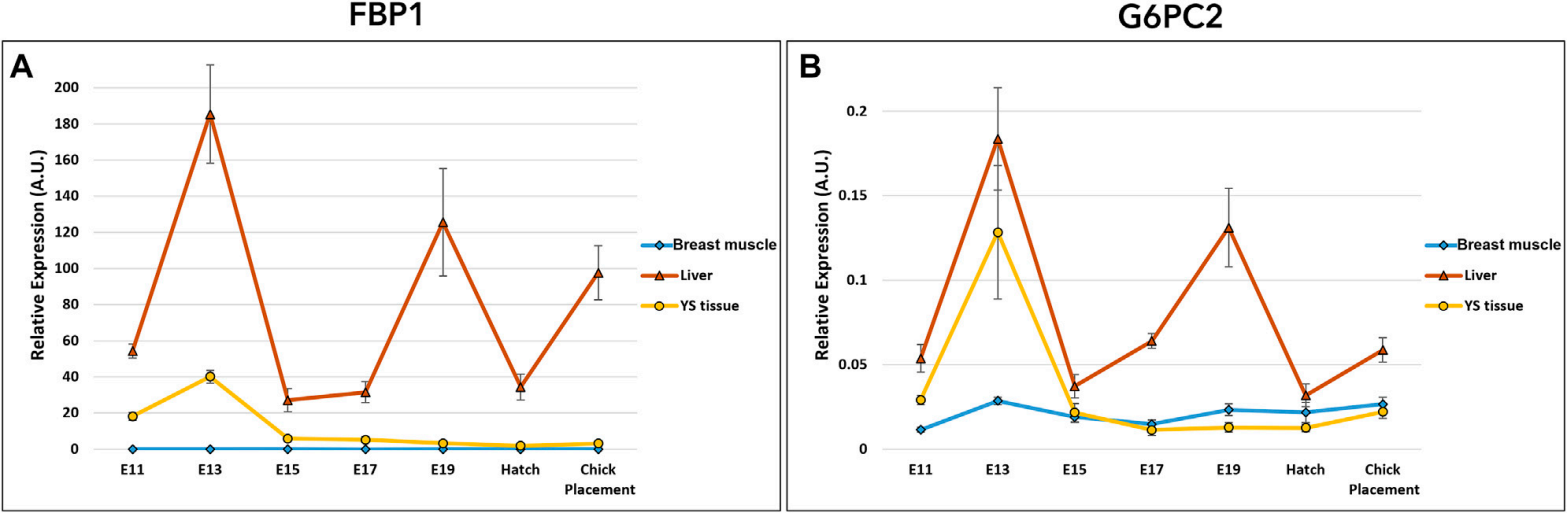
Dynamic expression of genes involved in gluconeogenesis **(A)** Fructose-1,6-bisphosphatase 1 (FBP1), regulatory enzyme, catalyzes the hydrolysis of fructose 1,6-bisphosphate to fructose 6-phosphate **(B)** Glucose-6- phosphatase 2 (G6PC2), catalyzes the hydrolysis of glucose-6-phosphate, allowing the release of glucose into the bloodstream. Expression analysis of FBP1 and G6PC2 by a two-way ANOVA followed by Tukey’s HSD test revealed significant interactions between the effects of age and tissue. (*p* < 0.05), *n* = 6 per day.

Gluconeogenesis potential was evaluated by the examination of two genes: fructose-1,6-bisphosphatase 1 (FBP1) and glucose-6-phosphatase 2 (G6PC2) (Figure 5 and Supplementary Table S4). Expression analysis of FBP1 and G6PC2 by a two-way ANOVA test revealed significant interactions between the effects of age and tissue (*p* < 0.0001). Also here, the interaction is associated with the altering expression patterns in tissues. Results showed daily fluctuation in liver FBP1 expression. In the YS tissue, FBP1 expression was elevated on E13 and significantly decreased on E15, then remained constant throughout incubation up to chick placement. In the breast muscle, FBP1 expression level was relatively low, decreasing between E15 and E17, and increasing again at placement. The variation in expression pattern of G6PC2 was similar to that of FBP1 expression in the liver, breast muscle and YS tissue. In general, when comparing the expression of gluconeogenic genes between the liver, YS tissue and breast muscle within days, the liver showed the highest values throughout incubation.

### Glycogen synthesis and breakdown in the liver, YS tissue, and breast muscle of chicken embryos

**FIGURE 6:**
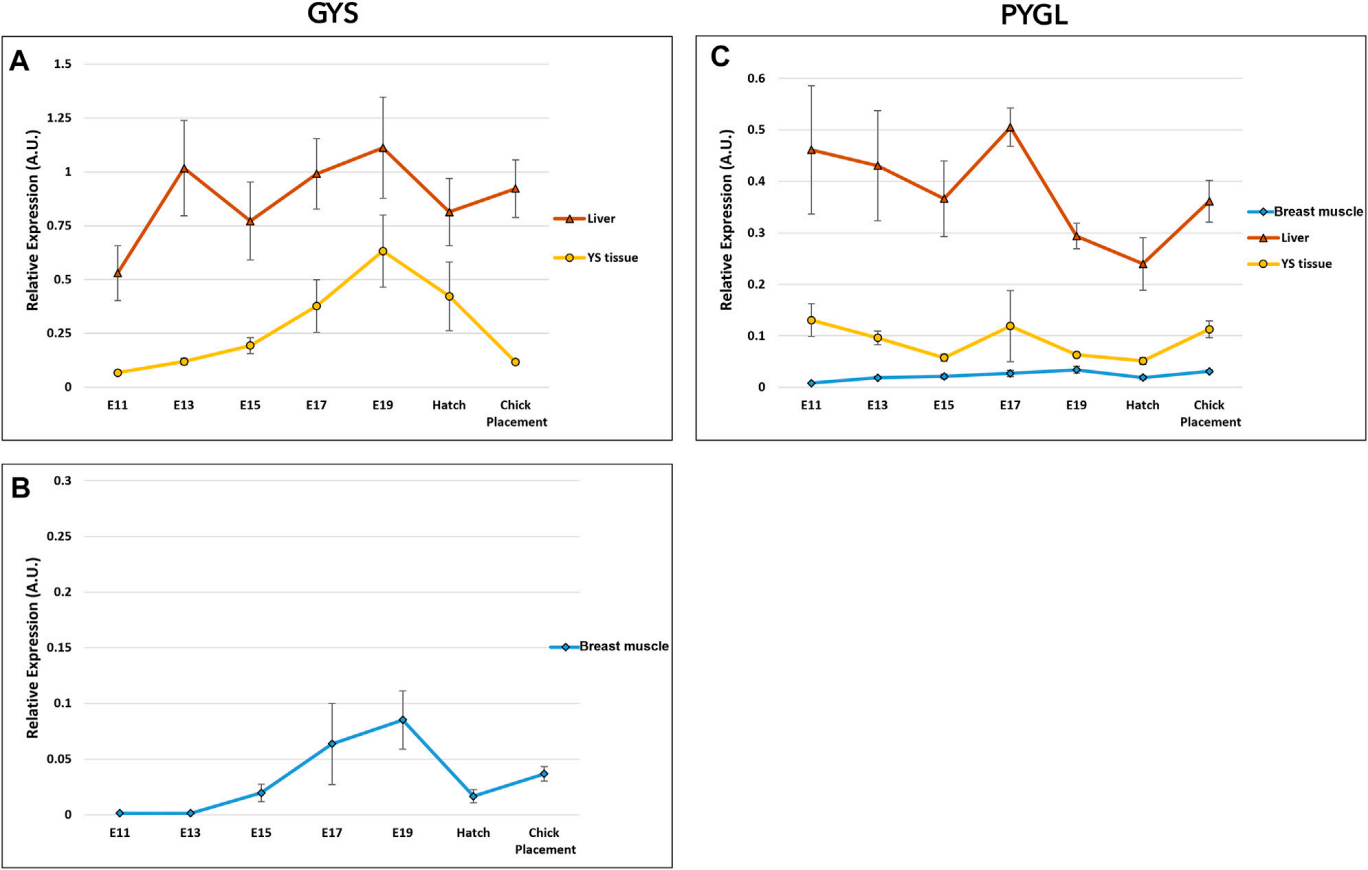
Dynamic expression of genes involved in glycogen synthesis and breakdown. **(A, B)** Glycogen synthase (GYS*) catalyzes the rate-limiting step in the synthesis of glycogen; **(C)** Glycogen phosphorylase L (PYGL), catalyzes the release of glucose- 1 -phosphate from glycogen. As derived from a two-way ANOVA followed by Tukey’s HSD test, the factor of tissue was found to be significantly different in GYS and PYGL (highest in the liver, mean in the YS tissue and lowest in the breast muscle). The factor age was also significant, as E 1 9 was highest in GYS and E17 was highest in PYGL (*p* < 0.05), *n* = 6 per day. *Two variants were used for glycogen synthase gene; **(A)** GYS2 in the liver and YS tissue and **(B)** GYS one in the breast muscle.

The potential of glycogen synthesis and breakdown was evaluated by two genes: glycogen synthase (GYS) and glycogen phosphorylase L (PYGL) (Figure 6 and Supplementary Table S5). Glycogen synthase (GYS) has two isoforms/variants: glycogen synthase 1 (GYS1) and GYS2 (Roach et al., 1998). GYS1 is expressed at lower levels in muscle, whereas GYS2 is primarily responsible for glycogen synthesis in the liver (Browner et al., 1989; Nuttall et al., 1994). Accordingly, in the breast muscle, the muscle variant GYS1 was used for expression analysis of glycogen synthase, while in the YS tissue and liver, GYS2 was used. As for, glycogen phosphorylase, the variant PYGL was used for the breast muscle, YS tissue and liver. PYGL was shown to be expressed at the protein level also in the breast muscle (Zambonelli et al., 2016). A two-way ANOVA test revealed that there was no significant interaction between the effects of age and tissue in GYS and PYGL expression. The tissue factor had statistically significant effect on GYS expression (*p* < 0.0001) with the highest expression levels in the liver, medium values in the YS tissue and lowest in the breast muscle. Similarly, the age factor was also found to be significant (*p* = 0.00035), with a peak in overall GYS expression at E19, significantly different from all sampling points apart from E17. As for PYGL, the tissue factor had a significant effect (*p* < 0.0001) with the highest expression levels in the liver, medium values in the YS tissue and lowest in the breast muscle. The age factor was also found to be significant (*p* = 0.049), with the highest PYGL expression at E17. As in the gluconeogenic genes, expression analysis of glycogen synthesis and breakdown genes between tissues within each day, showed the highest expression levels in the liver, with medium values in the YS tissue and lowest in the breast muscle.

## Discussion

The fertile egg contains trace amounts of available energy molecules in the form of carbohydrates (Yadgary and Uni, 2012; Réhault-Godbert et al., 2019) and creatine (Reicher et al., 2020). Accordingly, chicken embryos depend on the supply of high energy-value molecules that are synthesized, stored, and utilized by the embryonic and extraembryonic tissues. The current study investigated the dynamics of the two major energy-value molecules, creatine and glycogen, in key tissues for energy metabolism, from mid incubation (E11) until hatch and placement at the farm. Based on our findings, creatine is stored mainly in the breast muscle, while glycogen is stored mainly in the YS tissue. We demonstrate, for the first time, that creatine synthesis genes (AGAT, GAMT) are expressed in the chicken breast muscle as early as mid-incubation. The expression analysis of genes related to gluconeogenesis (FBP1, G6PC2), glycogenesis (GYS), and glycogenolysis (PYGL), reveals that the liver constitutes the main site of glycogen metabolism activity. However, due to the relatively large size and high storage capacity of the breast muscle and YS tissue, their contribution to glycogen metabolism is substantial. Towards hatch, creatine levels in all tissues increased, while glycogen levels decreased post E19 reaching low levels at hatch and at chick placement. Together, the findings suggest that when glycogen stores are depleted, creatine serves as an available energy source for late-term embryos and hatchlings.

In accordance with the traditional description of spatial separation between the supply and demand organs of endogenous creatine (Wyss, 2000), our results show that in chicken embryos creatine synthesis genes (AGAT, GAMT) are expressed in the liver and kidney. Here we show, that AGAT and GAMT are also expressed in the extraembryonic YS tissue. This fits the concept of the YS tissue as a multifunctional metabolic organ, which supports embryonic development (Yadgary et al., 2010; Yadgary et al., 2011; Yadgary and Uni, 2012; Yadgary et al., 2013; Yadgary et al., 2014; Dayan et al., 2020; Wong and Uni, 2021). To the best of our knowledge, we are the first to show AGAT and GAMT expression in the breast muscle of chicken embryos; the expression of these genes was previously found in the skeletal muscle of fish, mice, cattle, and pigs (Borchel et al., 2019). Ostojic (2021) points towards a metabolic advantage of *de novo* creatine synthesis in myocytes to maintain muscle creatine levels and cellular energy. The levels of creatine in the liver, YS tissue and breast muscle show similar pattern of increase up to hatch, where creatine is stored mainly in the breast muscle. Nevertheless, considering the relative size of organs, the significance of the YS tissue, the largest organ during incubation is highlighted as a superior creatine source vs the liver, with higher total creatine amount (19-fold higher on E17 and 7-fold higher at hatch). Together, we conclude that during incubation the supply of creatine depends on its *de novo* synthesis by the embryonic and extraembryonic tissues. As creatine levels increase towards hatch, it serves as an essential energy source, especially to late-term embryos and hatchlings.

Comparable patterns of glycogen levels were exhibited in the breast muscle, liver and YS tissue where all tissues reached peak values on E17-E19, followed by a sharp decrease towards chick placement. Previous studies showed a similar pattern corresponding with the changes in energy metabolism towards hatch and decreased glycogen levels in tissues (Christensen et al., 1999; Christensen et al., 2000; Christensen et al., 2001; Uni and Ferket, 2004; Uni et al., 2005; Foye et al., 2006; Moran 2007; De Oliveira et al., 2008; Yadgary and Uni, 2012). This study shows that throughout incubation, the majority of glycogen is stored in the YS tissue. This agrees with a previously published study by Yadgary and Uni (2012), which showed the importance of the YS tissue as the major glycogen storage organ. Our results suggest a pivotal role of the YS tissue, not only in creatine supply, but also in the supply of glycogen during mid-term incubation up to the initiation of the energy-demanding hatching process. Expression analysis of genes involved in glycogen synthesis and breakdown (GYS and PYGL, respectively), showed the highest expression levels in the liver compared with mid values in the YS tissue and the lowest values in the breast muscle. According to these findings, E19 marks a critical time point, showing a shift in balance between glycogen synthesis and breakdown. The decrease in GYS expression but not in PYGL expression fits the observed decrease in glycogen levels. On this day (E19), as oxygen becomes limited (Tazawa et al., 1983), other metabolic pathways are involved in glucose and ATP production, shifting towards anaerobic catabolism of glucose, with the breakdown of glycogen and gluconeogenesis (Uni and Ferket, 2004; Moran, 2007; De Oliveira et al., 2008; Yadgary and Uni 2012). Indeed, the increase in ketones levels up to E17 correspond with β-oxidation of yolk-derived fatty acids, and the rise in blood glucose levels (starting on E19) correspond with the metabolic shift and the decrease in tissue glycogen levels from E17 onwards. At hatch, when oxygen supply was restored, ketones levels increase again. The expression of the two key genes FBP1 and G6PC2 was the highest in the liver along the incubation period with mid values in the YS tissue and lowest in the breast muscle. Together, our results lead to the conclusion that during the second half of incubation the gluconeogenic pathway as well as glycogen synthesis and breakdown is most active in the liver. However, due to the relatively large size and high storage capacity of the breast muscle and YS tissue, their contribution to energy supply for the chicken embryo should not be neglected.

**FIGURE 7:**
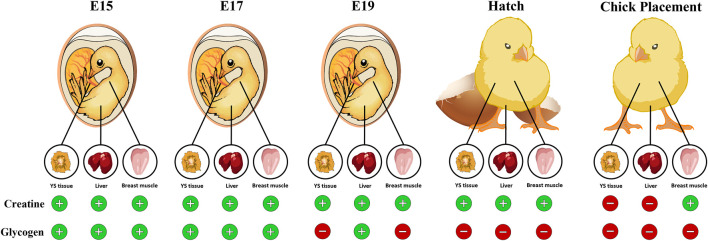
Graphical illustration demonstrating the available energy resources (creatine and glycogen) in the chicken embryo during the pre-post hatch period. Towards hatch, creatine levels in the YS tissue, liver and breast muscle increase while glycogen levels dramatically decrease, reaching low levels at hatch and at chick placement. In the breast muscle, as glycogen levels are depleted, creatine levels are elevated. Thus, highlighting the importance of creatine as an available energy source for the late-term embryo and hatchling.

In conclusion, this study evaluated the dynamics of creatine, glycogen, and their synthesis during the second half of the broiler embryonic development period. We demonstrate that during incubation, creatine is stored mainly in the breast muscle while glycogen is stored mainly in the YS tissue. The exhibited decrease in glycogen levels towards hatch in all tissues, starting on E19, opposes the increase in creatine levels, suggesting the significance of creatine in energy supply for late-term embryos and hatchlings (summarized in Figure 7). Moreover, in breast muscle, as glycogen levels are depleted, high creatine levels are maintained until chick placement, emphasizing the utmost importance of creatine involvement in supplying available energy for the hatching chick before placement at the farm. The fact that creatine is involved in maintaining available energy supply for the hatching broiler chick could have a great impact when considering hatchling quality, uniformity, chick survival and post-hatch performance.

## Data Availability

The original contributions presented in the study are included in the article/Supplementary Materials, further inquiries can be directed to the corresponding author.
