# Supplementary material for: Supply and demand of creatine and glycogen in broiler chicken embryos
Source: Front Physiol. 2023 Jan 24;14:1079638. doi: 10.3389/fphys.2023.1079638 (PMC9902709; doi:10.3389/fphys.2023.1079638)
Supplement: Supplementary file 1 [file DataSheet1.PDF]

SUPPLEMENTARY MATERIAL

Supply and demand of creatine and glycogen in broiler chicken embryos

Jonathan Dayan[1], Tal Melkman-Zehavi[1], Naama Reicher[1], Ulrike Braun[2], Vivienne Inhuber[2], Sameer J. Mabjeesh[1], Orna Halevy[1], Zehava Uni[1*]

[1]Department of Animal Science, Robert H. Smith Faculty of Agriculture, Food and Environment, The Hebrew University of Jerusalem, Rehovot 7610001, Israel

[2]AlzChem Trostberg GmbH, Dr.-Albert-Frank-Straße 32, 83308 Trostberg, Germany

Supplementary Table 1. Creatine and glycogen concentration

	Creatine dry weight concentration (mg/g)			Glycogen dry weight concentration (mg/g)		
	Breast muscle	Liver	YS tissue	Breast muscle	Liver	YS tissue
**E11**	-	0.03 ± 0.02 ^{b}	0.13 ± 0.01 ^{abc}	-	27.98 ± 4.41 ^{c}	24.02 ± 2.66 ^{bc}
**E13**	-	0.05 ± 0.01 ^{b}	0.05 ± 0.004 ^{d}	-	36.09 ± 1.52 ^{bc}	15.21 ± 1.96 ^{bc}
**E15**	5.93 ± 0.56 ^{b}	0.09 ± 0.01 ^{ab}	0.07 ± 0.01 ^{cd}	15.86 ± 1.18 ^{b}	59.48 ± 3.55 ^{ab}	24.98 ± 2.75 ^{bc}
**E17**	8.72 ± 0.47 ^{a}	0.1 ± 0.02 ^{ab}	0.12 ± 0.02 ^{abc}	20.23 ± 0.60 ^{a}	79.78 ± 10.11 ^{a}	43.63 ± 4.89 ^{a}
**E19**	7.53 ± 0.32 ^{ab}	0.13 ± 0.04 ^{ab}	0.13 ± 0.02 ^{ab}	17.68 ± 0.45 ^{ab}	79.87 ± 13.36 ^{a}	25.99 ± 4.63 ^{bc}
**Hatch**	7.18 ± 0.42 ^{ab}	0.19 ± 0.05 ^{a}	0.17 ± 0.02 ^{a}	13.85 ± 0.87 ^{bc}	27.33 ± 6.98 ^{bc}	16.42 ± 2.48 ^{bc}
**Chick placement**	8.52 ± 0.74 ^{a}	0.09 ± 0.01 ^{ab}	0.11 ± 0.01 ^{bcd}	9.78 ± 1.49 ^{c}	2.93 ± 2E-16 ^{c}	11.46 ± 2.54 ^{c}

Dry weight concentration (mg/g) of breast muscle, liver, and yolk sac (YS) tissue from E11 until hatch and chick placement. The lower-case letters denote for means significantly different between days within tissues, as derived from a one-way ANOVA Tukey's HSD test. ($P \leq 0.05$), n=6 per day.

Supplementary Table 2. Creatine and glycogen amount per tissue

	Total creatine amount (mg)			Total glycogen amount (mg)		
	Breast muscle	Liver	YS tissue	Breast muscle	Liver	YS tissue
**E11**	-	0.001 ± 0.0005 **G**	0.10 ± 0.01 **FG**	-	0.69 ± 0.14 **G**	18.60 ± 2.95 **EFG**
**E13**	-	0.003 ± 0.001 **G**	0.08 ± 0.01 **FG**	-	2.17 ± 0.31 **FG**	26.74 ± 4.54 **DE**
**E15**	0.71 ± 0.05 **D**	0.009 ± 0.001 **G**	0.20 ± 0.03 **EFG**	1.91 ± 0.04 **FG**	5.85 ± 0.46 **EFG**	68.62 ± 5.29 **B**
**E17**	1.25 ± 0.11 **C**	0.02 ± 0.004 **G**	0.39 ± 0.08 **DEF**	2.88 ± 0.18 **FG**	14.87 ± 1.69 **EFG**	127.75 ± 7.47 **A**
**E19**	1.54 ± 0.06 **BC**	0.034 ± 0.01 **G**	0.29 ± 0.06 **EFG**	3.62 ± 0.15 **FG**	21.07 ± 3.89 **DEF**	57.34 ± 11.32 **BC**
**Hatch**	1.59 ± 0.09 **B**	0.06 ± 0.014 **FG**	0.43 ± 0.04 **DE**	3.09 ± 0.25 **FG**	9.26 ± 2.48 **EFG**	40.63 ± 4.54 **CD**
**Chick placement**	2.33 ± 0.20 **A**	0.04 ± 0.006 **FG**	0.116 ± 0.013 **EFG**	2.69 ± 0.44 **FG**	1.24 ± 0.08 **FG**	13.10 ± 3.81 **EFG**

Total tissue amount (mg) of breast muscle, liver, and yolk sac (YS) tissue from E11 until hatch and chick placement. The capital letters denote for means significantly different between days and tissues, as derived from a two-way ANOVA followed by Tukey's HSD test. ($P \leq 0.05$), n=6 per day.

Supplementary Table 3. Expression of genes involved in creatine synthesis in the breast muscle, liver, YS tissue and kidney

	AGAT				GAMT			
	Breast muscle	Liver	YS tissue	Kidney	Breast muscle	Liver	YS tissue	Kidney
**E11**	0.72 ± 0.23 **F**	6.34 ± 1.64 **DEF**	3.08 ± 0.96 **EF**	1.99 ± 0.33 **F**	0.05 ± 0.007 **EF**	1.35 ± 0.22 **BCDEF**	0.15 ± 0.019 **EF**	0.43 ± 0.047 **EF**
**E13**	0.2 ± 0.08 **F**	3.51 ± 0.67 **EF**	3.12 ± 0.28 **EF**	0.57 ± 0.11 **F**	0.01 ± 0.003 **F**	0.2 ± 0.07 **EF**	0.21 ± 0.04 **EF**	0.24 ± 0.06 **EF**
**E15**	3.31 ± 0.49 **EF**	5.99 ± 2.67 **DEF**	5.95 ± 1.6 **DEF**	2.03 ± 0.34 **F**	0.099 ± 0.005 **EF**	0.98 ± 0.14 **CDEF**	1.86 ± 0.33 **ABCD**	0.533 ± 0.03 **DEF**
**E17**	9.21 ± 0.86 **CDE**	3.11 ± 0.37 **EF**	3.95 ± 1.55 **EF**	1.35 ± 0.34 **F**	0.25 ± 0.025 **EF**	1.36 ± 0.19 **BCDEF**	2.96 ± 0.52 **A**	0.535 ± 0.09 **DEF**
**E19**	13.96 ± 2.37 **BC**	3.35 ± 0.58 **EF**	1.59 ± 0.52 **F**	2.22 ± 0.54 **EF**	0.49 ± 0.08 **DEF**	1.46 ± 0.44 **BCDE**	2.11 ± 0.43 **ABC**	0.49 ± 0.1 **DEF**
**Hatch**	5.02 ± 0.41 **EF**	12.54 ± 1.64 **BCD**	0.83 ± 0.16 **F**	1.94 ± 0.98 **F**	0.18 ± 0.03 **EF**	1.38 ± 0.41 **BCDEF**	1.85 ± 0.49 **ABCD**	0.82 ± 0.28 **CDEF**
**Chick placement**	18.25 ± 2.83 **AB**	23.17 ± 3.48 **A**	0.12 ± 0.014 **F**	1.13 ± 0.204 **F**	0.35 ± 0.08 **EF**	2.41 ± 0.69 **AB**	0.31 ± 0.04 **EF**	0.89 ± 0.103 **CDEF**

Abbreviations: arginine-glycine amidinotransferase (AGAT), guanidinoacetate N-methyltransferase (GAMT). The capital letters denote for means significantly different between days and tissues, as derived from a two-way ANOVA followed by Tukey's HSD test. ($P \leq 0.05$), n=6 per day.

Supplementary Table 4. Expression of genes involved in gluconeogenesis in the breast muscle, liver and YS tissue

	FBP1			G6PC2		
	Breast muscle	Liver	YS tissue	Breast muscle	Liver	YS tissue
**E11**	0.01 ± 0.002 **E**	54.33 ± 3.88 **CD**	18.17 ± 2.16 **DE**	0.012 ± 0.0014 **C**	0.054 ± 0.008 **C**	0.03 ± 0.003 **C**
**E13**	0.018 ± 0.0015 **E**	185.33 ± 27.16 **A**	40.15 ± 3.52 **DE**	0.029 ± 0.002 **C**	0.18 ± 0.03 **A**	0.13 ± 0.04 **AB**
**E15**	0.02 ± 0.004 **E**	27.05 ± 6.4 **DE**	5.79 ± 1.57 **E**	0.019 ± 0.0035 **C**	0.04 ± 0.007 **C**	0.02 ± 0.0035 **C**
**E17**	0.007 ± 0.0007 **E**	31.44 ± 5.85 **DE**	5.18 ± 1.57 **E**	0.015 ± 0.003 **C**	0.064 ± 0.0044 **BC**	0.011 ± 0.003 **C**
**E19**	0.009 ± 0.003 **E**	125.52 ± 29.6 **B**	3.18 ± 0.75 **E**	0.023 ± 0.0024 **C**	0.13 ± 0.023 **A**	0.0128 ± 0.003 **C**
**Hatch**	0.008 ± 0.003 **E**	34.42 ± 7.18 **DE**	1.82 ± 0.22 **E**	0.02 ± 0.0035 **C**	0.03 ± 0.006 **C**	0.126 ± 0.0022 **C**
**Chick placement**	0.019 ± 0.005 **E**	97.64 ± 14.91 **BC**	3.07 ± 0.74 **E**	0.027 ± 0.004 **C**	0.058 ± 0.007 **C**	0.022 ± 0.004 **C**

Abbreviations: fructose-1,6-bisphosphatase 1 (FBP1), glucose-6-phosphatase 2 (G6PC2). The capital letters denote for means significantly different between days and tissues, as derived from a two-way ANOVA followed by Tukey's HSD test. ($P \leq 0.05$), n=6 per day.

Supplementary Table 5. Expression of genes involved in glycogen synthesis and breakdown in the breast muscle, liver and YS tissue

	GYS			PYGL		
	Breast muscle	Liver	YS tissue	Breast muscle	Liver	YS tissue
**E11**	0.00013 ± 0.0004	0.53 ± 0.13	0.07 ± 0.006	0.008 ± 0.001	0.46 ± 0.12	0.13 ± 0.03
**E13**	0.00014 ± 0.0002	1.02 ± 0.22	0.12 ± 0.015	0.02 ± 0.004	0.43 ± 0.1	0.096 ± 0.013
**E15**	0.02 ± 0.008	0.77 ± 0.18	0.19 ± 0.036	0.021 ± 0.004	0.37 ± 0.07	0.058 ± 0.007
**E17**	0.06 ± 0.03	0.99 ± 0.16	0.38 ± 0.12	0.027 ± 0.006	0.5 ± 0.04	0.12 ± 0.07
**E19**	0.085 ± 0.026	1.11 ± 0.23	0.63 ± 0.17	0.03 ± 0.006	0.29 ± 0.025	0.06 ± 0.005
**Hatch**	0.017 ± 0.006	0.81 ± 0.16	0.42 ± 0.16	0.019 ± 0.004	0.24 ± 0.05	0.05 ± 0.006
**Chick placement**	0.037 ± 0.007	0.92 ± 0.13	0.11 ± 0.011	0.03 ± 0.003	0.36 ± 0.04	0.11 ± 0.016

Abbreviations: glycogen synthase muscle variant (GYSM), glycogen synthase (GYS2), glycogen phosphorylase L (PYGL). As derived from a two-way ANOVA followed by Tukey's HSD test, the factor of tissue was found to be significantly different in GYS and PYGL (highest in the liver, mean in the YS tissue and lowest in the breast muscle). The factor age was also significant, as E19 was highest in GYS and E17 was highest in PYGL. ($P \leq 0.05$), n=6 per day.

Supplementary Table 6. Embryo and tissue weights

	Embryo YFBM (g)	Yolk content (g)	Breast muscle		Liver		YS tissue	
			Weight (g)	% Of YFBM	Weight (g)	% Of YFBM	Weight (g)	% Of YFBM
**E11**	5.51 ± 0.23	20.95 ± 1.17	0.22 ± 0.01	3.97 ± 0.29	0.10 ± 0.005	1.82 ± 0.09	2.23 ± 0.05	40.6 ± 1.09
**E13**	12.7 ± 0.46	14.71 ± 0.81	0.65 ± 0.03	5.09 ± 0.24	0.25 ± 0.01	2.0 ± 0.07	4.07 ± 0.2	32.06 ± 1.27
**E15**	21.37 ± 0.71	10.4 ± 0.72	1.09 ± 0.05	5.11 ± 0.19	0.41 ± 0.013	1.89 ± 0.05	5.73 ± 0.27	26.89 ± 1.48
**E17**	28.82 ± 0.7	8.65 ± 1.11	1.11 ± 0.05	3.86 ± 0.11	0.67 ± 0.02	2.33 ± 0.08	6.79 ± 0.58	23.86 ± 2.57
**E19**	39.81 ± 0.34	4.65 ± 0.37	1.33 ± 0.07	3.35 ± 0.17	0.82 ± 0.02	2.05 ± 0.06	6.22 ± 0.22	15.63 ± 0.59
**Hatch**	44.9 ± 0.43	3.22 ± 0.39	1.47 ± 0.03	3.28 ± 0.07	1.06 ± 0.012	2.36 ± 0.02	5.18 ± 0.26	11.52 ± 0.52
**Chick placement**	44.23 ± 0.47	0.81 ± 0.36	1.77 ± 0.07	4.01 ± 0.18	1.27 ± 0.05	2.87 ± 0.12	2.78 ± 0.16	6.27 ± 0.34

Wet weight (g) of embryo yolk free body mass (YFBM), yolk content, breast muscle, liver, and YS tissue and the relative weights of breast muscle, liver, and YS tissue (% of YFBM).

(A) **Effect Tests**

Source	Nparm	DF	Sum of Squares	F Ratio	Prob > F	
Organ	2	1	0.8611991	35.3424	<.0001*	LostDFs
Age	6	4	2.7001800	27.7029	<.0001*	LostDFs
Organ*Age	12	10	6.0463741	24.8135	<.0001*	LostDFs

Least Squares Means Plot

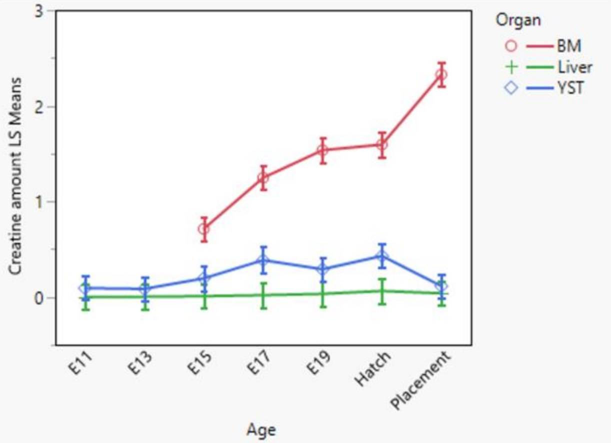

Level								Least Sq Mean
BM,Placement	A							2.3268997
BM,Hatch		B						1.5945695
BM,E19		B	C					1.5362660
BM,E17			C					1.2481822
BM,E15				D				0.7133431
YST,Hatch				D	E			0.4299332
YST,E17				D	E	F		0.3862488
YST,E19					E	F	G	0.2905785
YST,E15					E	F	G	0.1951065
YST,Placement					E	F	G	0.1155643
YST,E11						F	G	0.0939062
YST,E13						F	G	0.0856795
Liver,Hatch						F	G	0.0633602
Liver,Placement						F	G	0.0392313
Liver,E19							G	0.0338151
Liver,E17							G	0.0197185
Liver,E15							G	0.0089870
Liver,E13							G	0.0034882
Liver,E11							G	0.0007713
BM,E11	A	B	C	D	E	F	G	0.0000000
BM,E13				D	E	F	G	0.0000000

Levels not connected by same letter are significantly different.

(B) **Effect Tests**

Source	Nparm	DF	Sum of Squares	F Ratio	Prob > F	
Organ	2	1	37387.508	412.4918	<.0001*	LostDFs
Age	6	4	17102.345	47.1720	<.0001*	LostDFs
Organ*Age	12	10	28621.564	31.5778	<.0001*	LostDFs

Least Squares Means Plot

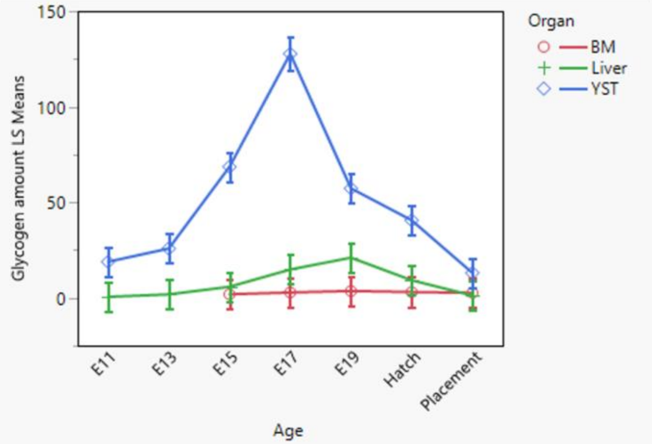

Level								Least Sq Mean
YST,E17	A							127.75387
YST,E15		B						68.62291
YST,E19		B	C					57.33551
YST,Hatch			C	D				40.63259
YST,E13				D	E			25.84582
Liver,E19				D	E	F		21.06884
YST,E11					E	F	G	18.92079
Liver,E17					E	F	G	14.86712
YST,Placement					E	F	G	13.09845
Liver,Hatch					E	F	G	9.26059
Liver,E15					E	F	G	5.85061
BM,E19						F	G	3.62098
BM,Hatch						F	G	3.09499
BM,E17						F	G	2.88278
BM,Placement						F	G	2.68543
BM,E15						F	G	1.90916
Liver,E13						F	G	1.88028
Liver,Placement						F	G	1.23882
Liver,E11							G	0.58148
BM,E11	A	B	C	D	E	F	G	0.00000
BM,E13				D	E	F	G	0.00000

Levels not connected by same letter are significantly different.

Supplementary Figure 1. Summary of parameter estimates as derived from a two-way ANOVA followed by Tukey's HSD test for (A)creatine and (B)glycogen total amount in the breast muscle(BM), liver and YS tissue(YST). Significant interactions were found between the effects of age and tissue.

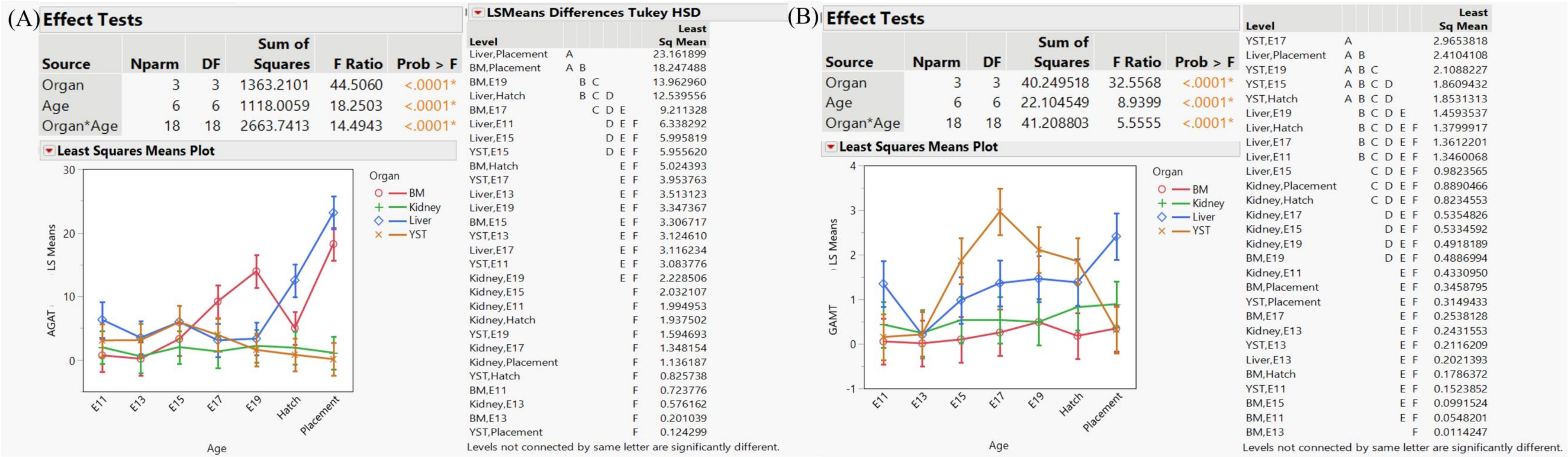

(A) **Effect Tests**

Source	Nparm	DF	Sum of Squares	F Ratio	Prob > F
Organ	3	3	1363.2101	44.5060	<.0001*
Age	6	6	1118.0059	18.2503	<.0001*
Organ*Age	18	18	2663.7413	14.4943	<.0001*

LSMeans Differences Tukey HSD

Level		Least Sq Mean
Liver,Placement	A	23.161899
BM,Placement	A B	18.247488
BM,E19	B C	13.962960
Liver,Hatch	B C D	12.539556
BM,E17	C D E	9.211328
Liver,E11	D E F	6.338292
Liver,E15	D E F	5.995819
YST,E15	D E F	5.955620
BM,Hatch	E F	5.024393
YST,E17	E F	3.953763
Liver,E13	E F	3.513123
Liver,E19	E F	3.347367
BM,E15	E F	3.306717
YST,E13	E F	3.124610
Liver,E17	E F	3.116234
YST,E11	E F	3.083776
Kidney,E19	E F	2.228506
Kidney,E15	F	2.032107
Kidney,E11	F	1.994953
Kidney,Hatch	F	1.937502
YST,E19	F	1.594693
Kidney,E17	F	1.348154
Kidney,Placement	F	1.136187
YST,Hatch	F	0.825738
BM,E11	F	0.723776
Kidney,E13	F	0.576162
BM,E13	F	0.201039
YST,Placement	F	0.124299

Levels not connected by same letter are significantly different.

(B) **Effect Tests**

Source	Nparm	DF	Sum of Squares	F Ratio	Prob > F
Organ	3	3	40.249518	32.5568	<.0001*
Age	6	6	22.104549	8.9399	<.0001*
Organ*Age	18	18	41.208803	5.5555	<.0001*

Level		Least Sq Mean
YST,E17	A	2.9653818
Liver,Placement	A B	2.4104108
YST,E19	A B C	2.1088227
YST,E15	A B C D	1.8609432
YST,Hatch	A B C D	1.8531313
Liver,E19	B C D E	1.4593537
Liver,Hatch	B C D E F	1.3799917
Liver,E17	B C D E F	1.3612201
Liver,E11	B C D E F	1.3460068
Liver,E15	C D E F	0.9823565
Kidney,Placement	C D E F	0.8890466
Kidney,Hatch	C D E F	0.8234553
Kidney,E17	D E F	0.5354826
Kidney,E15	D E F	0.5334592
Kidney,E19	D E F	0.4918189
BM,E19	D E F	0.4886994
Kidney,E11	E F	0.4330950
BM,Placement	E F	0.3458795
YST,Placement	E F	0.3149433
BM,E17	E F	0.2538128
Kidney,E13	E F	0.2431553
YST,E13	E F	0.2116209
Liver,E13	E F	0.2021393
BM,Hatch	E F	0.1786372
YST,E11	E F	0.1523852
BM,E15	E F	0.0991524
BM,E11	E F	0.0548201
BM,E13	F	0.0114247

Levels not connected by same letter are significantly different.

Supplementary Figure 2. Summary of parameter estimates as derived from a two-way ANOVA followed by Tukey's HSD test for the expression of genes involved in creatine synthesis; (A) AGAT and (B) GAMT, in the breast muscle(BM), liver, YS tissue(YST) and kidney. Significant interactions were found between the effects of age and tissue.

(A) **Effect Tests**

Source	Nparm	DF	Sum of Squares	F Ratio	Prob > F
Organ	2	2	152462.42	150.7184	<.0001*
Age	6	6	56270.57	18.5423	<.0001*
Organ*Age	12	12	72718.44	11.9811	<.0001*

Least Squares Means Plot

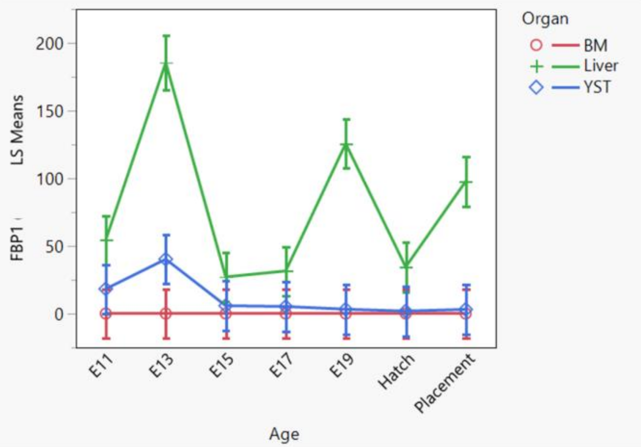

Level					Least Sq Mean
Liver,E13	A				185.34986
Liver,E19		B			125.52372
Liver,Placement		B	C		97.64378
Liver,E11			C	D	54.33180
YST,E13				D E	40.15106
Liver,Hatch				D E	34.41810
Liver,E17				D E	31.44072
Liver,E15				D E	27.05512
YST,E11				D E	18.16943
YST,E15				E	5.79029
YST,E17				E	5.18148
YST,E19				E	3.17702
YST,Placement				E	3.06897
YST,Hatch				E	1.81663
BM,E15				E	0.02122
BM,Placement				E	0.01849
BM,E13				E	0.01764
BM,E11				E	0.01271
BM,E19				E	0.00854
BM,Hatch				E	0.00792
BM,E17				E	0.00727

Levels not connected by same letter are significantly different.

(B) **Effect Tests**

Source	Nparm	DF	Sum of Squares	F Ratio	Prob > F
Organ	2	2	0.08082072	40.2674	<.0001*
Age	6	6	0.11080365	18.4019	<.0001*
Organ*Age	12	12	0.06591869	5.4738	<.0001*

Least Squares Means Plot

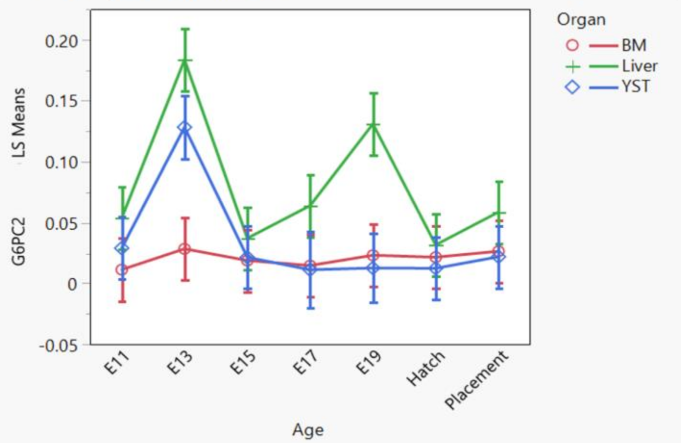

Level				Least Sq Mean
Liver,E13	A			0.18368146
Liver,E19	A			0.13107420
YST,E13	A	B		0.12838793
Liver,E17		B	C	0.06401875
Liver,Placement			C	0.05871553
Liver,E11			C	0.05376952
Liver,E15			C	0.03722789
Liver,Hatch			C	0.03179907
YST,E11			C	0.02912872
BM,E13			C	0.02855943
BM,Placement			C	0.02656392
BM,E19			C	0.02328941
YST,Placement			C	0.02206447
BM,Hatch			C	0.02170143
YST,E15			C	0.02161813
BM,E15			C	0.01899884
BM,E17			C	0.01485267
YST,E19			C	0.01283006
YST,Hatch			C	0.01257374
BM,E11			C	0.01149848
YST,E17			C	0.01136643

Levels not connected by same letter are significantly different.

Supplementary Figure 3. Summary of parameter estimates as derived from a two-way ANOVA followed by Tukey's HSD test for the expression of genes involved in gluconeogenesis; (A) FBP1 and (B) G6PC2, in the breast muscle(BM), liver and YS tissue(YST). Significant interactions were found between the effects of age and tissue.

(A) **Effect Tests**

Source	Nparm	DF	Sum of Squares	F Ratio	Prob > F
Organ	2	2	15.989284	95.3544	<.0001*
Age	6	6	1.756057	3.4908	0.0035*
Organ*Age	12	12	1.184319	1.1771	0.3090

Level				Least Sq Mean
Liver	A			0.87956826
YST		B		0.27581061
BM			C	0.03211324

Levels not connected by same letter are significantly different.

Level			Least Sq Mean
E19	A		0.60966299
E17	A	B	0.47737625
Hatch	A	B	0.41768002
E13	A	B	0.37942635
Placement	A	B	0.35875980
E15	A	B	0.32830355
E11		B	0.19960596

Levels not connected by same letter are significantly different.

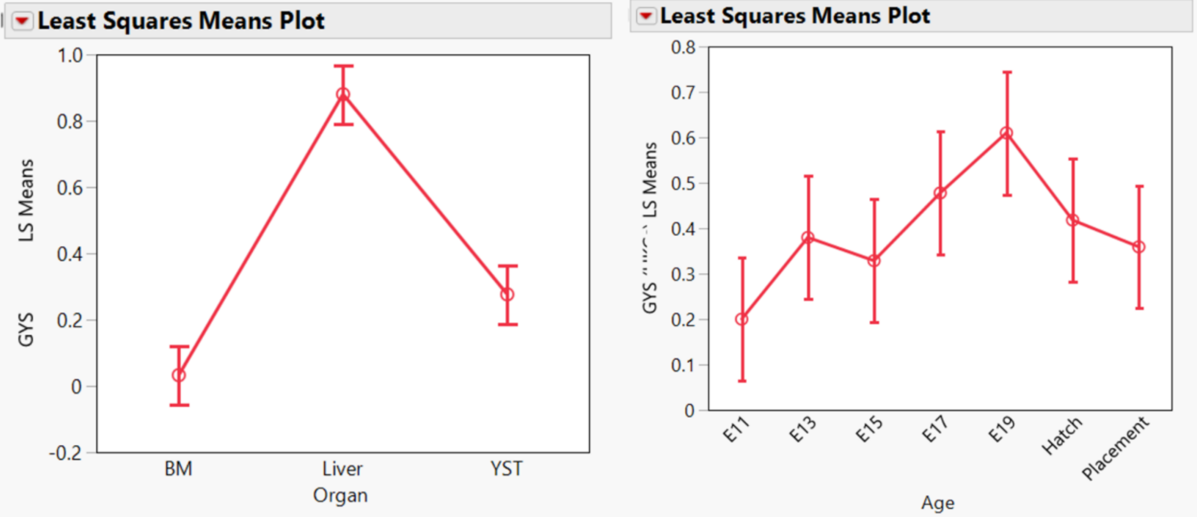

(B) **Effect Tests**

Source	Nparm	DF	Sum of Squares	F Ratio	Prob > F
Organ	2	2	3.0112208	118.5697	<.0001*
Age	6	6	0.1668021	2.1893	0.0499*
Organ*Age	12	12	0.1835226	1.2044	0.2902

Level				Least Sq Mean
Liver	A			0.37987711
YST		B		0.09010476
BM			C	0.02280782

Levels not connected by same letter are significantly different.

Level		Least Sq Mean
E17	A	0.21712893
E11	A	0.20005549
E13	A	0.18194521
Placement	A	0.16849394
E15	A	0.14845688
E19	A	0.13042418
Hatch	A	0.10333797

Levels not connected by same letter are significantly different.

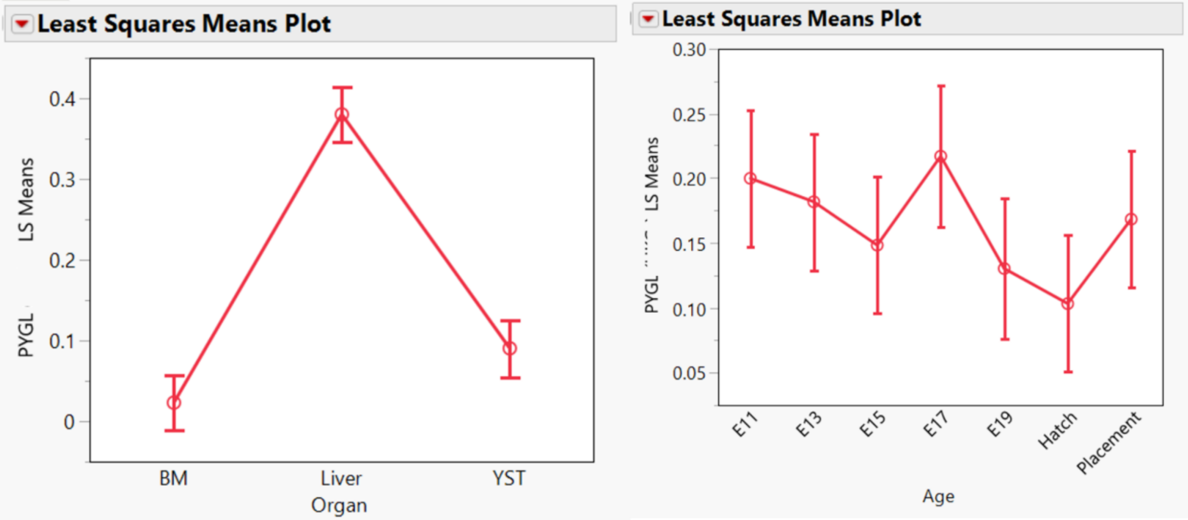

Supplementary Figure 4. Summary of parameter estimates as derived from a two-way ANOVA for the expression of genes involved in (A) glycogen synthesis (GYS) and (B) breakdown (PYGL) in the breast muscle(BM), liver and YS tissue(YST). The factors age and tissue were found to be significant in GYS and PYGL.
